# Gastroenterological Manifestations of Immunoglobulin G Subclass 4-Related Disease—Epidemiology, Clinical Manifestations, Diagnosis and Treatment

**DOI:** 10.3390/life13081725

**Published:** 2023-08-11

**Authors:** Jorge Lucas de Sousa Moreira, Sarah Maria Bacurau Barbosa, Pedro Lucas Gomes Moreira de Meneses, Pedro Garcia Dias de Barros, Samuel de Sá Barreto Lima, Damiao Maroto Gomes Junior, Gledson Micael da Silva Leite, Jacob Oliveira Duarte, Galba Matos Cardoso de Alencar Junior, Maria Auxiliadora Ferreira Brito Almino, José Matos Cruz, Hermes Melo Teixeira Batista, Estelita Lima Cândido, Gislene Farias de Oliveira, Hellen Lúcia Macedo Cruz, Jucier Gonçalves Júnior

**Affiliations:** 1 School of Medicine, Universidade Federal do Cariri (UFCA), Barbalha 63180-000, CE, Brazilsarah.bacurau@aluno.ufca.edu.br (S.M.B.B.); pedro.meneses@aluno.ufca.edu.br (P.L.G.M.d.M.); pedro.garcia@aluno.ufca.edu.br (P.G.D.d.B.); sa_barreto@globomail.com (S.d.S.B.L.); jacob.duarte@ufca.edu.br (J.O.D.); maria-auxiliadora.brito@ufca.edu.br (M.A.F.B.A.);; 2 Programa de Pós-Graduação em Ciências da Saúde, School of Medicine, Universidade Federal do Cariri (UFCA), Barbalha 63180-000, CE, Brazil; junior.maroto@aluno.ufca.edu.br (D.M.G.J.); gledson.micael@aluno.ufca.edu.br (G.M.d.S.L.); 3 School of Dentistry, CECAPE, Juazeiro do Norte 63024-015, CE, Brazil; galbaalencar2@hotmail.com; 4 Programa de Pós-Graduação em Ciências da Saúde, Universidade Federal de Sergipe (UFS), Aracaju 49032-490, SE, Brazil; 5 Programa de Pós-Graduação em Biotecnologia em Saúde Humana e Animal, Universidade Estadual do Ceará (UECE), Fortaleza 60356-000, CE, Brazil; 6 Department of Rheumatology, Faculdade de Medicina da Universidade de São Paulo (FMUSP), São Paulo 01246-903, SP, Brazil

**Keywords:** diagnosis, immunoglobulin G4-related disease, gastrointestinal tract, epidemiology, systematic review

## Abstract

Despite causing high morbidity, IgG4-related disease (IgG4-RD) and its gastroenterological manifestations lack better and greater theoretical contributions. Therefore, the objective of this work was to evaluate the clinical–epidemiological, diagnostic and treatment aspects of the gastrointestinal manifestations of this disease. A systematic review was carried out using the PubMed, Scopus and Embase databases between January 2012 and March 2023 with the following descriptors: “Immunoglobulin G4-Related Disease” (MeSH) AND #2 “Gastrointestinal Tract” (MeSH). Our data collection grouped a total of 3607 patients from mostly epidemiological cohort studies and cross-sectional follow-ups. In the subgroup analysis, IgG4-RD was associated with male gender, with an estimated prevalence between 54% and 80%. In our findings, the prevalence by topography was presented in the following ranges: lacrimal glands (17–57%); salivary glands (28–72%); pancreas (19–60%); biliary tract (5–40%); retroperitoneal cavity (9–43%). Longitudinal studies are needed to better map the natural history of the gastrointestinal manifestations of IgG4-RD and enable the formulation of individualized treatments.

## 1. Introduction

Immunoglobulin G subclass 4-related disease (IgG4-RD) is a chronic, multisystemic, idiopathic disease characterized by a lymphoplasmacytic inflammatory process that leads to fibrosis of organs and systems. Historically, IgG4-RD was described as a heterogeneous set of diseases with an emphasis on gastrointestinal involvement, especially pancreatitis. Its characterization came from histopathological and immunological studies and mapping of the response to immunosuppressive agents [1,2,3].

Due to the heterogeneous clinical spectrum of gastroenterological manifestations of IgG4-RD, the diagnosis is challenging, requiring a correlation between clinical, laboratory and imaging aspects [4,5,6]. This is concerning, as the literature indicates the involvement of two or more organs simultaneously or recurrently in more than 75% of patients with IgG4-RD. In contrast, the gastrointestinal system is affected in 60–87% of cases. The most frequent associations are autoimmune pancreatitis (AIP) and sclerosing cholangitis. However, lacrimal, salivary and thyroid glands as well as the stomach, intestines, kidneys and retroperitoneum can also be affected [2,3,4].

Despite the high morbidity [3] and the complexity of the diagnosis [5,6] the literature still lacks more and better theoretical contributions on this topic. Thus, the objective of this study was to carry out a systematic review of the literature to map clinical, epidemiological and therapeutic aspects of the gastrointestinal manifestations of IgG4-RD.

## 2. Materials and Methods

### 2.1. Literature Review

A qualitative systematic review of the literature was conducted following the PRISMA protocol. Electronic databases including PubMed, Scopus, ScienceDirect (Elsevier) and the Virtual Health Library (VHL) were searched using the following strategy: #1 “Immunoglobulin G4-Related Disease” (MeSH) AND #2 “Gastrointestinal Tract” (MeSH) between January 2012 and June 2023. The year 2012 was chosen as the starting point because it marked the publication of the disease description.

The study was carried out with the acronym PICOS, with “P” being patients with IgG4-RD, “I” being the clinical–epidemiological characterization of the gastrointestinal manifestations of IgG4-RD, “C” being the healthy control group and “O” being the outcomes of the gastrointestinal manifestations of IgG4-RD.

### 2.2. Data Collection

Data collection took place in June 2023. The articles were pre-analyzed from their titles and abstracts. Two researchers collected data individually, with a third senior researcher responsible for evaluating discrepancies and doubts. After this selection, each article was read in full. The most relevant data from each article are given in Table 1, including the authors, objectives, results and main findings. The main findings include inherent clinical, epidemiological, diagnostic and therapeutic aspects of the gastrointestinal manifestations of IgG4-RD.

To analyze the quality of each study, for the convenience of the authors, the Study Quality Assessment tool (https://www.nhlbi.nih.gov/health-topics/study-quality-assessment-tools, accessed on 15 June 2023) created by the National Heart, Lung and Blood Institute (NHLB) was used. Cohort studies were evaluated using the Quality Assessment Tool for Observational Cohort and Cross-Sectional Studies and case–control studies were evaluated using the Quality Assessment of Case–Control Studies. These tools classify studies as “good”, “fair” or “poor” based on the presence or absence of relevant methodological elements for each type of study.

### 2.3. Eligibility Criteria

Articles in English, Portuguese and Spanish that were original, complete and related to the subject of the study were selected. Inclusion criteria considered suitability for the purpose of this review; availability and transparency of data; methodological rigor applied with an emphasis on clinical, comparative and observational studies; number of participants greater than one hundred (*n* > 100); and prevalence of the topography of involvement due to IgG4-RD. Review articles, brief comments, editorials, communications and letters to the editor were excluded.

### 2.4. Ethical Issue

Considering that this is a systematic literature review, Resolution 510/16 of the Brazilian National Health Council (CNS, acronym in Portuguese) dismissed the requirement for approval from a Human Research Ethics Committee.

## 3. Results

**Figure 1:**
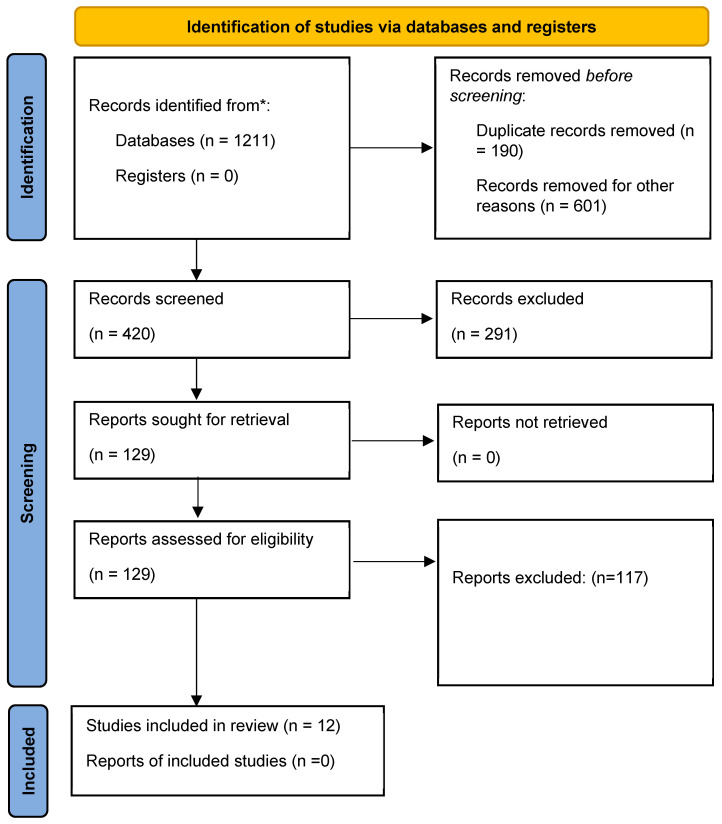
Identification flowchart of included studies. * number of papers found in the four databases.

Initially, 1211 articles were identified in the databases searched (150 from PubMed, 485 from Scopus, 564 from ScienceDirect and 12 from VHL). After exclusion by title and abstract, 227 articles were selected for full-text analysis (Figure 1). In the end, twelve articles met the eligibility criteria (Table 1).

Most (91.2%) of the sample comprised cohort studies, while the rest were case–control studies. As for the quality analysis, 41.66% were classified as good, 41.66% as fair and 16.68% as poor.

For heuristic reasons, the data were divided as follows: “epidemiology of the gastrointestinal manifestations of IgG4-RD”, “clinical and diagnostic aspects of the gastrointestinal manifestations of IgG4-RD” and “therapeutic approach to the gastrointestinal manifestations of IgG4-RD”.

### 3.1. Epidemiology of Gastrointestinal Manifestations of IgG4-RD

According to our search strategy, 3607 patients had gastrointestinal manifestations of IgG4-RD, and the majority were Asian (mainly Japanese), with a follow-up period between 4 and 5 years of the disease. Survival rates ranged from 95.3% to 89.0% for 5 to 10 years of disease, respectively [2,19,20]. According to literature data, the prevalence of IgG4-RD gastrointestinal manifestations is estimated to be 62 million people or 0.28–1.08 per 100,000 inhabitants between 2003 and 2009 [2]. However, more recent studies point to an increase in these indicators to 2.2/100,000 inhabitants [19].

This fact is intriguing as it is not commonly observed in other autoimmune diseases such as systemic lupus erythematosus, rheumatoid arthritis, systemic sclerosis, autoimmune myopathies and sarcoidosis, among others. This could be interesting to consider in the clinical reasoning for the differential diagnoses between the gastrointestinal involvement of IgG4-RD and connective tissue diseases.

Individuals affected by IgG4-RD often have other comorbidities, particularly type 2 diabetes mellitus and allergic diseases, such as rhinosinusitis, bronchial asthma and drug allergies [9]. Furthermore, the literature suggests that smoking is associated with a greater chance of having IgG4-RD, primarily among patients with retroperitoneal fibrosis attributed to IgG4-RD, those with normal serum concentrations of IgG4 and female patients [21].

### 3.2. Clinical and Diagnostic Aspects of the Gastrointestinal Manifestations of IgG4-RD

**Figure 2:**
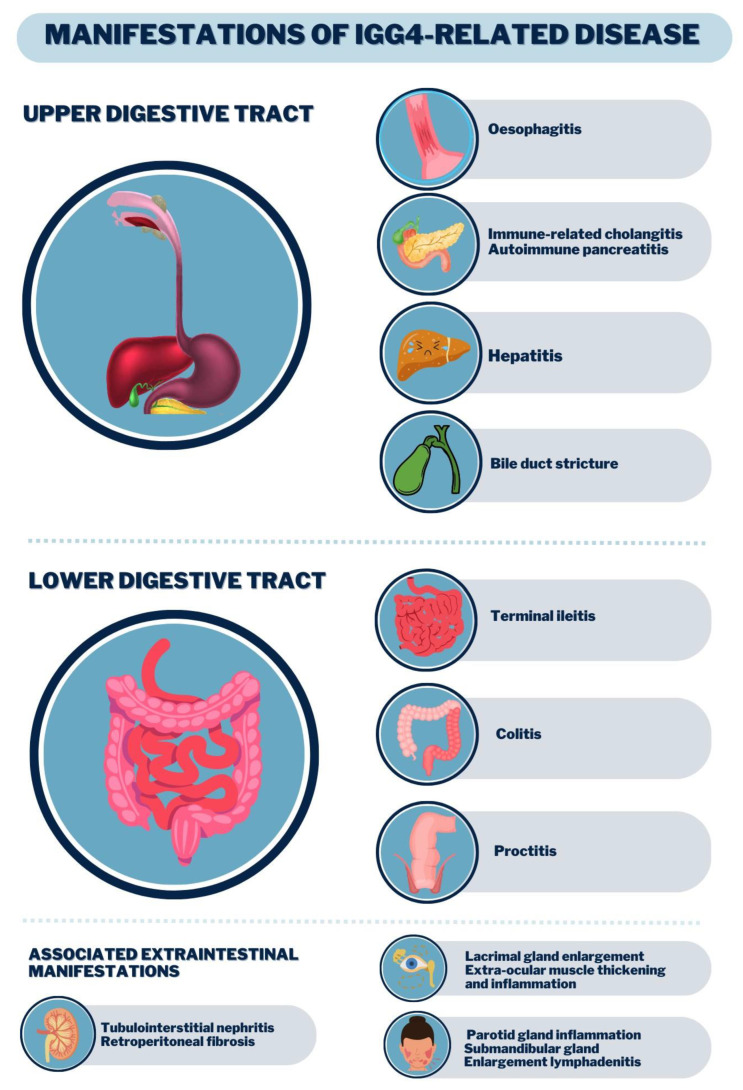
Clinical manifestations of the digestive tract by IgG4-RD.

IgG4-RD is progressive, insidious leading to organ dysfunction or mass effects. It commonly affects glands (e.g., lacrimal, major salivary, pancreas, thyroid), the biliary tract, retroperitoneum, lungs, aorta, kidneys, meninges and orbits, and the concomitant involvement of these structures is a strong indication of IgG4-RD [1]. It is subdivided into four clusters: (a) limited to the head and neck, (b) pancreaticobiliary, (c) aorto-peritoneal and (d) systemic Mikulicz, whose involvement is metachronous; that is, as one organ experiences a regression of symptoms, another is affected [2,3,5]. Organ involvement is shown in Figure 2. Diagnostic criteria for IgG4-RD are listed in Table 2.

The main laboratory changes in IgG4-RD are (1) normocytic–normochromic anemia with eosinophilia; (2) increase in immunoglobulin levels such as IgG and IgG4, with (3) polyclonal peak in serum protein electrophoresis; (4) increase in C-reactive protein (CRP) and erythrocyte sedimentation volume; (5) when certain organs are affected, an increase in alkaline phosphatase, gamma-glutamyl transferase (in autoimmune pancreatitis and primary sclerosing cholangitis), salivary amylase (in parotitis) and complement consumption (C3 and C4) may be seen in renal dysfunction [1,2,3,4,5].

Regarding the pathophysiology of IgG4-RD, the most widely accepted theory is that there is a relationship between environmental factors (e.g., infections with *Helicobacter pylori*, *Mycobacterium tuberculosis* and Gram-negative bacteria) and immunological factors (e.g., stimulation of Toll-like receptors) that trigger an immune response at the expense of Th-2 lymphocytes, with an increase in interleukins (IL) such as IL-4, IL-5 and IL-13. This immune response is accompanied by the proliferation and maturation of eosinophils, the recruitment of macrophages and differentiation of B lymphocytes, the consumption of complement (C3 and C4) and the increased production of IgG4. The increase in IgG4 is also considered an epiphenomenon. Subsequently, tissue infiltration with lymphoplasmacytic infiltrate occurs, leading to tumors and/or tissue destruction [5].

In general, the diagnosis of IgG4-RD is one of exclusion (Table 2). A biopsy [8,14,15] of the affected tissue should be performed, detecting lymphoplasmacytic infiltrate with immunophenotyping for IgG4 [15] with the presence of positive IgG4 cells above the limit of 50% of the field of view under the microscope and IgG4/IgG ratio above 40%. A classic appearance of the infiltrate in IgG4-RD is the storiform appearance typified by the “wagon wheel” appearance of the fibroblast arrangement [9,12,25]. Serum IgG4 levels may be elevated, but this is not pathognomonic (Table 2).

Imaging studies are commonly used in cases of autoimmune pancreatitis due to IgG4-RD with contrast-enhanced computed tomography, nuclear magnetic resonance and endoscopic ultrasound exams [9,12,25]. For pathologies restricted to the head and neck, ultrasound is also a valuable tool for differential diagnosis [1]. Upper digestive endoscopy and/or colonoscopy can be useful for biopsy and evaluation of possible associated pathologies in the esophagus, stomach and intestines [8,10,11,14,15].

**Figure 3:**
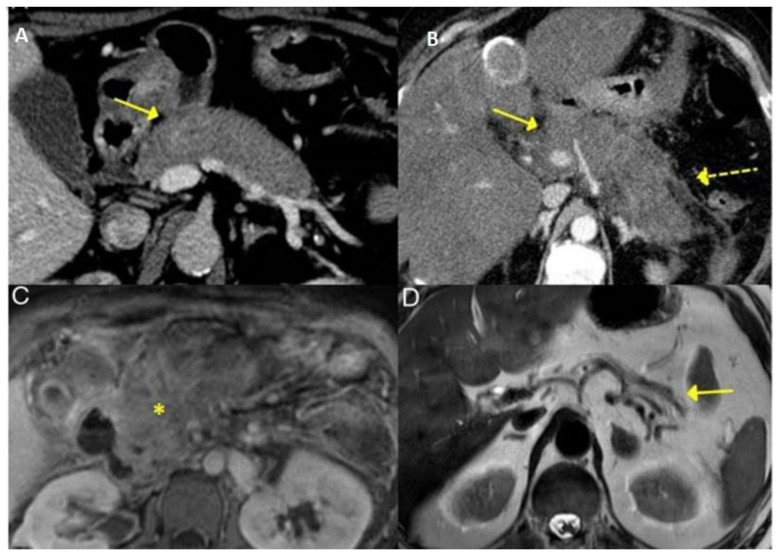
Typical features of AIP 2 in IgG4-RD seen on CT and MRI—Diffuse enlargement of the pancreas at CT (**A**,**B**) with blurring of peripancreatic fat (dashed yellow arrow, on **B**). T1-weighted MRI image of the pancreas (**C**, asterisk indicates pancreatic fibrosis) showing pancreatic atrophy (solid yellow arrow, **D**) [Adapted] [23].

**Figure 4:**
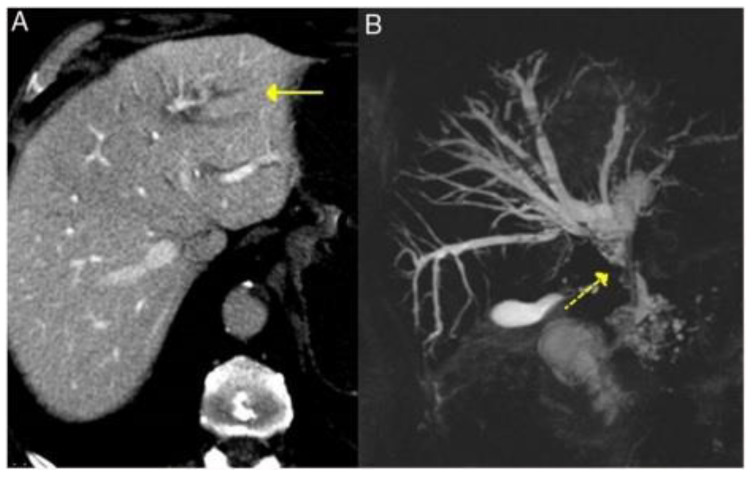
Typical features of primary sclerosing cholangitis in IgG4-RD seen on CT and MRI—dilatation of the intrahepatic bile ducts on the left side (solid arrow, **A**) and cholangio-MRI image showing dilatation of the extrahepatic and intrahepatic bile ducts (left and right) with areas of stenosis (dashed yellow arrow, **B**) [Adapted] [23].

**Figure 5:**
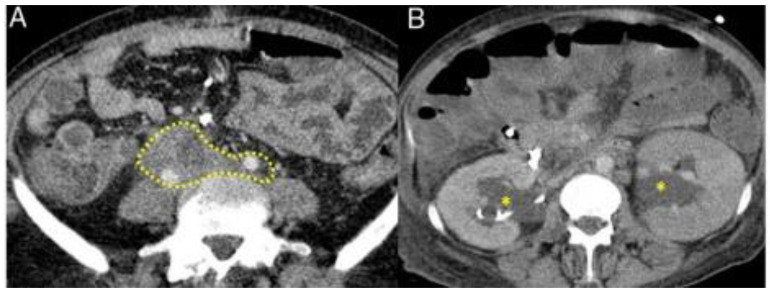
Typical features of retroperitoneal fibrosis in IgG4-RD seen on CT and MRI. Fibrotic process involving the ureters and iliac arteries in the abdomen (dashed mark, **A**) with hydronephrosis (asterisks, **B**). [Adapted] [23].

The CT and MRI aspects of the most common involvement of IgG4-RD in the gastrointestinal tract are shown in Figure 3, Figure 4 and Figure 5.

Next, there is a characterization by topographical anatomy of clinical aspects of the different manifestations of IgG4-RD in the gastrointestinal system.

#### 3.2.1. Head and Neck

Glandular involvement was commonly observed in most studies, with the salivary glands and eyes being most commonly affected. When these manifestations are associated with systemic signs and symptoms, the diagnosis time is one year; however, if they occur in isolation, this time doubles [10,11]. The main symptoms associated with head and neck disorders include edema with increased growth and swelling of the lacrimal/submandibular and parotid glands and chronic rhinosinusitis [8,14,15].

#### 3.2.2. Esophagus, Stomach and Intestines

Involvement of the esophagus, stomach and intestine in IgG4-RD is rare. The few descriptions in the literature indicate the presence of high serum levels of IgG4 and chronic esophagitis with the presence of conduction dysphagia, heartburn, regurgitation and retrosternal pain refractory to the usual treatment for gastroesophageal reflux disease [9].

Furthermore, elevated serum IgG4 levels are rarely observed in the intestine. Most cases are based primarily on an increase in IgG4-positive cells, often without reported IgG4/IgG ratios. Therefore, there is still significant uncertainty regarding the involvement of organs such as the esophagus, stomach and intestine in IgG4-related disease [12].

#### 3.2.3. Pancreas

The pancreas may present type 1 and 2 autoimmune pancreatitis (AIP 1 and 2). The major pancreatic manifestation of IgG4-RD is type 1 autoimmune pancreatitis (AIP). In type 1 AIP, the clinical manifestations can be acute (obstructive jaundice and/or pancreatic mass) or chronic (pancreatic atrophy, calcification and ductal dilation). The differentiation between them is a challenge for three reasons: (i) imaging tests (e.g., endoscopic ultrasound, MRI and CT with contrast) may show similar characteristics, including parenchymal enlargement, sometimes resembling a sausage shape, peripancreatic edematous borders and narrowing of the main pancreatic duct without upstream dilation (Figure 3); (ii) response to treatment with glucocorticoids is common for both pathologies; (iii) both are associated with pathologies such as inflammatory bowel disease, although IBD is more commonly linked to type 2. AIP 2 or idiopathic granulocytic or central ductal autoimmune pancreatitis has no specific serologic markers (e.g., IgG4 levels are normal); it usually has no systemic manifestations because it is a disease of the pancreas alone. Therefore, no other organs are affected. It occurs earlier than AIP 1 and is more common in men [25].

Three aspects can be used to differentiate type 1 and 2 AIPs: (i) serological abnormalities; (ii) serum IgG4 levels; (iii) histopathological analysis. Histopathological analysis demonstrates a pattern of lymphoplasmacytic infiltration affecting the pancreatic tissue in a diffuse or irregular manner, storiform fibrosis and phlebitis obliterans. Finally, it is possible to observe an increase in serum levels of IgG4 in cases of 1 AIP [8,25].

#### 3.2.4. Liver and Bile Ducts

Also known as immunoglobulin G4-related hepatobiliary disease, this condition manifests as glucocorticoid-responsive cholangitis of the extrahepatic and perihilar bile ducts, often associated with type 1 AIP. The symptomatology presents as jaundice, pruritus, weight loss and abdominal pain [16]. However, inflammatory pseudotumors of the liver and biliary cirrhosis can also develop as some late manifestations of this condition [9,15].

There is an elevation in serum markers of cholestasis, including alkaline phosphatase, gamma-glutamyl transferase and conjugated bilirubin. Histopathological criteria include lymphoplasmacellular infiltrates with more than 10 IgG4-positive plasma cells in the area visible under the microscope, storiform fibrosis and phlebitis obliterans. Minor criteria include eosinophilia and partial obliterative phlebitis [15].

Cholangiographic characteristics are classified into five subtypes based on the involvement of the biliary tree: (i) inferior stenosis of the distal bile duct, (ii) segmental or (iii) diffuse intrahepatic stenosis, (iv) minor stenosis of the distal bile duct or (v) a combination of hilar and inferior stenoses of the distal bile duct or isolated hilar stenoses. The use of therapeutic response to corticosteroids as a diagnostic criterion is controversial [9]. The CT and MRI aspects are shown in Figure 4.

#### 3.2.5. Retroperitoneum

Retroperitoneal fibrosis (RPF) is the process of fibro-inflammation in the peri-aortic and peri-iliac retroperitoneum (Figure 5). It is a condition that affects adjacent structures and causes ureteral obstruction, with post-renal acute kidney injury being one of the most common complications, followed by glandular and pancreatic involvement. As for the prevalent signs and symptoms, edema of glands such as the pancreas and prostate, nonspecific low back pain or diffuse abdominal pain, malaise, anorexia, edema in the lower limbs, fever and weight loss can be highlighted [11,13,17].

Regarding renal involvement, the disease is manifested by typical clinical characteristics such as uremic syndrome or elevation of nitrogenous slags. In histopathological terms, there are lesions similar to tumors, dense infiltration with IgG4-positive plasmocytes and extensive fibrosis. In laboratory terms, it is important to highlight that it is the only manifestation of IgG4-RD that involves complement consumption (C3 and C4). This type of condition is usually related to diagnostic delays that allow the accumulation of involvement in additional organs and increase the risk of permanent damage to these organs [7,18].

### 3.3. Therapeutic Approach to the Gastrointestinal Manifestations of IgG4-RD

Treatment for IgG4-RD depends on the degree of disease activity, the affected organs and the patient’s symptomatology. In the case of gastrointestinal symptoms, the therapy of choice is glucocorticoid, at a dose of 0.6 to 0.8 mg/kg per day orally for 1 month, aiming to induce remission. Then, the dose should be gradually reduced by 5 mg every 2 weeks, evaluating the disease activity clinically and through laboratory tests during follow-up [25].

Glucocorticoid therapy proved to be the most effective for initial control of the disease, achieving response rates of 97–100%. However, there are not enough studies demonstrating its effectiveness as a maintenance treatment. Even so, about 33% of patients will relapse during weaning from corticosteroid therapy, requiring the reintroduction of the loading dose. There are no known effective means of preventing IgG4-RD relapse. There are recommendations for low-dose maintenance corticosteroid therapy that can last for several years, capable of reducing this chance from 58% to 23%. Furthermore, immunosuppressants can also be used, especially to induce remission in refractory cases, but the combined use with glucocorticoids is initially not a unanimous recommendation [2,4].

Currently, in patients with recurrent IgG4-RD, high-dose glucocorticoids can be used, followed or not by rituximab. This biologic is used at a dose of 375 mg/m^2^ of body surface area, weekly, for 4 weeks, followed by infusions every 2–3 months. Studies with immunosuppressants such as azathioprine (2–3 mg/Kg/day), mycophenolate mofetil (2–3 g/day), methotrexate (15–25 mg/week) and calcineurin inhibitors are insufficient. However, it is known that relapses are common when using these medications in medical practice [2,4]. In general, patients showed a good response to treatment, but the response was incomplete in long-lasting lesions, which can be explained by the prominent fibrotic component of the lesion [25,26].

#### 3.3.1. Head and Neck

In the case of involvement of the lacrimal and salivary glands in the head and neck region, treatment usually involves the use of glucocorticoids, such as prednisone. This medication is administered in gradually reduced doses over time, with the aim of reducing the inflammatory process and relieving symptoms. In some more severe cases or refractory to glucocorticoids, immunosuppressants such as azathioprine and rituximab may be used [8].

In specific cases with obstruction of the lacrimal and salivary glands, additional procedures may be required to relieve the obstruction and restore glandular function. This may include performing endoscopic procedures, such as dilation or placement of prostheses, to open obstructed lacrimal or salivary pathways [15].

#### 3.3.2. Oral Cavity, Esophagus, Stomach and Intestines

For the treatment of involvement of the oral cavity, esophagus, stomach and intestines, glucocorticoids are also the first line of treatment. In more severe cases or when symptoms do not respond adequately to glucocorticoids, azathioprine and methotrexate may be considered. When there is an obstruction or stenosis of the digestive tract, it may be necessary to perform dilation or endoscopic clearance [9,12].

#### 3.3.3. Pancreas

Type 1 AIP has a good response to initial glucocorticoid therapy, yet has high relapse rates. In addition to immunosuppressive therapy, the assessment of nutritional deficiencies is part of the approach, such as (i) liposoluble vitamins (A, D, E and K) and (ii) trace elements such as zinc, calcium and magnesium. Surgery is generally not indicated for patients with AIP, but may be considered when suspected pancreatic cancer cannot be excluded after diagnostic evaluation. It is important to remember that endocrine insufficiency or pancreatic duct stones may be present, reinforcing the need for longitudinal follow-up in these cases [25,26].

#### 3.3.4. Liver and Bile Ducts

Involvement of the liver and biliary tract in IgG4-RD can lead to serious complications such as secondary sclerosing cholangitis (SSC). SSC treatment aims to control inflammation and prevent disease progression. The most commonly used medications are glucocorticoids, such as prednisone. In cases of recurrence or relapse, azathioprine, methotrexate or rituximab can be used. In severe cases of SSC with significant obstruction of the bile ducts, surgical intervention or endoscopic procedures to clear bile flow may be performed [9,16,25].

#### 3.3.5. Retroperitoneum

As in the other organs, in the retroperitoneum, glucocorticoids are first-line drugs followed by immunosuppressive therapy with azathioprine or rituximab in refractory or recurrent cases. A surgical approach or endoscopic procedures may be necessary in specific situations, such as when there is compression of adjacent structures or obstruction of vital organs such as post-renal acute kidney injury. These interventions are aimed at relieving the obstruction and restoring normal function [13,17,25].

## 4. Discussion

In the analysis of subgroups, IgG4-RD was associated with the male gender with an estimated prevalence ranging from 54 to 80%, while only 20–42% of the involvements were related to females. These results strengthen the association, consistency and analogy to the Bradford–Hill criteria. The literature indicates male predominance in these manifestations, typically occurring between the ages of 50 and 80, except for head and neck involvement. The disease rarely begins before the age of 40, with less than 10% of cases occurring in this age group [19,20,27].

It is important to understand that most studies are retrospective and were conducted in Asian populations (Table 1). Therefore, the data must be carefully analyzed for extrapolation to other ethnic groups, and it should be noted that retrospective studies are subject to bias.

Regarding the gastrointestinal manifestations of IgG4-RD, this review found that the most common involvement was observed in the lacrimal glands (17–57%) and salivary glands (28–72%), followed by pancreatic lesions (19–60%), the biliary tract (5–40%) and the retroperitoneum (9–43%). Most of these manifestations were diagnosed postoperatively as a presumed tumor [25,26] (Figure 2). According to literature data, there are at least two major clinical syndromes in IgG4-RD: jaundice and abdominal pain. Among the main signs and symptoms, we can highlight weight loss, pruritus, the appearance of abdominal masses in the topography of the pancreas or liver, and even mimicking neoplasms [2,5].

The diagnosis is clinical (Table 2), and treatment includes the use of glucocorticoids as the first choice, followed by other immunosuppressants (e.g., azathioprine, mofetil mycophenolate) or biologics such as rituximab.

It must be taken into account that the samples in the studies are small (Table 1). Although care was taken in this review to select only papers with *n* > 100, papers with large samples are difficult because IgG4-RD is a rare rheumatic disease. Consequently, data analysis is limited in terms of natural history and treatment approach. In addition, the difficulty of conducting longitudinal and prospective studies should be emphasized.

## 5. Conclusions

More than half of IgG4-RD gastrointestinal manifestations occur in men around the fifth decade of life. The commonly affected organs are the head and neck glands, the pancreas and the hepatobiliary tract. The diagnosis is one of exclusion, with biopsy being the gold standard.

There is still a scarcity in the literature to define a better immunosuppressive approach. As limitations of the present study, we can mention (i) the small number of selected articles and, consequently, of samples; (ii) the mostly Asian population; and (iii) data heterogeneity. However, it should be noted that these are elements inherent to the available literature. Thus, longitudinal studies are needed to better map the natural history of gastrointestinal manifestations of IgG4-RD and to allow the development of individualized treatments.

## Figures and Tables

**Table 1:** Summary of selected articles with emphasis on aspects of prevalence by gender and location of involvement.

Authors, Year, Study Design	Quality Analysis	Number of Participants	Purpose/Primary Outcome	Prevalence by Sex	Prevalence by Topographic Diagnosis	Main Findings
Wang et al., 2018, Cohort [7]	Regular	403	To evaluate how the difference between genders influences the treatment and prognosis of IgG4-RD	Symptoms and diagnosis begin primarily in females	Lacrimal glands: 40.2% Salivary glands: 45.4% Pancreas: 36.2% Biliary ducts: 21.6% Retroperitoneal cavity: 19.1%	The sex disparities in clinical characteristics of IgG4-RD indicated that male sex was independently associated with worse prognosis in response to glucocorticoid-based therapy.
Yamada et al., 2017, Cohort [8]	Good	334	Better and more broadly characterize clinical and laboratory aspects of IgG4-RD	Male: 61.4% Female: 38.6%	Lacrimal glands: 57.1% Salivary glands: 72.7% Pancreas: 25.5% Biliary ducts: 5.4% Retroperitoneal cavity: 24.9%	The serum CRP level is generally low and the serum IgG4 level is elevated in most Japanese IgG4-RD patients, in contrast to Western patients.
Inoue et al., 2015, Cohort [9]	Regular	235	Characterize the demographic profile and characteristics of patients with IgG4-RD	Male: 80% Female: 20%	Lacrimal glands: 23% Salivary glands: 34% Pancreas: 60% Biliary tracts: 0% Kidneys: 23% Retroperitoneal cavity: 20%	The IgG4 value was significantly higher in patients with multiorgan involvement than in those with a single manifestation (median 629 mg/dL vs. 299 mg/dL, *p* < 0.01). Of 218 patients, for whom both IgG4 and IgG values were available, the IgG4/IgG ratio was raised to >10% in 194 patients (89%).
Wallace et al., 2015, Cohort [10]	Good	125	Evaluate the correlation between clinical manifestations in patients diagnosed strictly by anatomopathological correlation	Male: 61% Female: 39%	Lacrimal glands: 22% Salivary glands: 28% Pancreas: 19% Biliary ducts: 0% Retroperitoneal cavity: 18%	Nearly 50% of this patient cohort with biopsy-proven, clinically active IgG4-RD had normal serum IgG4 concentrations. Elevations in the serum IgG4 concentration appeared to identify a subset of patients with a more severe disease phenotype.
Li et al., 2017, Cohort [11]	Fair	104	To review clinical features, treatment practices and factors associated with multisystem involvement of IgG4-RD in Hong Kong	Not specified	Lacrimal glands: 0% Salivary glands: 33% Pancreas: 40% Biliary tracts: 40% Retroperitoneal cavity 0%	Pre-treatment serum IgG4 is associated with multisystem disease, especially with salivary gland involvement, highlighting its potential for disease prognostication and monitoring.
Niwamoto et al., 2020, Cohort [12]	Regular	108	Classify IgG4-RD by a combination pattern of affected organs and identify their clinical characteristics, including the comorbidities of each subgroup	Male: 73% Female: 27%	Lacrimal glands: 31% Salivary glands: 68% Pancreas: 32% Biliary tracts: 0% Kidneys: 15% Retroperitoneal cavity: 22%	IgG4-RD can be classified into subgroups according to the pattern of affected organs. Group 5 may have frequent complications with allergies and malignancies.
Liu et al., 2023, Cohort [13]	Good	875	Externally validate the 2019 American College of Rheumatology and European League Against Rheumatism classification criteria for IgG4-RD and compare them to the 2020 RCD	Male: 59% Female: 41%	Lacrimal glands: 46% Salivary glands: 51% Pancreas: 37% Biliary ducts: 18% Retroperitoneal cavity: 14%	The 2019 ACR/EULAR classification criteria for IgG4-RD show outstanding specificity and good sensitivity in real-world clinical practice. The 2020 criteria are helpful for the diagnosis of IgG4-RD in clinical scenarios where IgG4-RD presents as involving an isolated organ, especially the unusual sites.
Liu et al., 2020, Cohort [14]	Good	407	To compare demographic, clinical and laboratory characteristics of IgG4-RD patients with retroperitoneal lesions	Male: 54% Female: 47%	Lacrimal glands: 55% Salivary glands: 72.2% Pancreas: 36.4% Biliary tracts: 18.1% Retroperitoneal cavity 9.1%	Demographic, clinical and laboratory differences between IgG4-RD RPF+ and RPF- patients indicated potential differences in pathogenesis and important implications for the diagnosis and management of these two phenotypes.
Zhang et al., 2017, Cohort [15]	Fair	346	Analyze clinical features of IgG4-RD to improve understanding of IgG4-RD in China	Male: 66.5% Female: 33.5%	Lacrimal glands: 46.5% Salivary glands: 52.6% Pancreas: 38.4% Biliary ducts: 25.4% Lung: 28% Retroperitoneal cavity: 19.9%	IgG4-RD is a systemic fibro-inflammatory disease with multiple-organ involvement. The most commonly involved organs include lymph nodes, submandibular glands and pancreas. Glucocorticoids and immunosuppressive agents were effective for IgG4-RD.
Zeng et al., 2021, Case–Control [16]	Good	450	To compare demographic, clinical and laboratory characteristics between IgG4-related kidney disease and extrarenal IgG4-related disease, as well as to describe radiological and pathological features	Male: 58% Female: 42%	Lacrimal glands: 48.4% Salivary glands: 65.6% Pancreas: 33.3% Biliary ducts: 0% Retroperitoneal cavity: 14.75%	It was found that renal function was impaired in approximately 40% of IgG4-RKD+ patients. The most common imaging finding is multiple, often bilateral, hypodense lesions. Male sex, more than three organs involved and low serum C3 level were risk factors for IgG4-RKD+ in IgG4-RD patients.
Lin et al., 2015, Cohort [17]	Regular	118	Characterize the clinical features of IgG4-RD	Male: 69% Female: 31%	Lacrimal glands: 0% Salivary glands: 64.4% Pancreas: 38.1% Biliary ducts: 17.8% Lung: 27.1% Retroperitoneal cavity: 26.3%	IgG4-RD is a systemic inflammatory and sclerosing disease. Parotid and lacrimal involvement (formerly called Mikulicz’s disease), lymphadenopathy and pancreatitis are the most common manifestations.
Zongfei et al., 2020, Cohort [18]	Regular	102	Identify predictive factors for treatment resistance and IgG4-RD relapse	Male: 75% Female: 25%	Lacrimal glands: 17.6% Salivary glands: 10.7% Pancreas: 20.5% Biliary ducts: 11.7% Retroperitoneal cavity: 43.1%	IgG4-RD is a systemic inflammatory and sclerosing disease. Parotid and lacrimal involvement (formerly called Mikulicz’s disease), lymphadenopathy and pancreatitis are the most common manifestations.

Legend: ACR: American College of Rheumatology; EULAR: European Alliance of Association for Rheumatology; IgG4-RD: IgG4-related disease; RCD: revised comprehensive diagnostic; RKD: related kidney disease; RPF: retroperitoneal fibrosis.

**Table 2:** The 2020 revised comprehensive diagnostic (RCD) criteria for IgG4-RD. Adapted from [22,23].

**[Item 1] Clinical and radiological features**
One or more organs show diffuse or localized swelling or a mass or nodule characteristic of IgG4-RD. In single-organ involvement, lymph node swelling is omitted.
**[Item 2] Serological diagnosis**
Serum IgG4 levels greater than 135 mg/dL.
**[Item 3] Pathological diagnosis**
**Positivity for two of the following three criteria:**
ο ① Dense lymphocyte and plasma cell infiltration with fibrosis.
ο ② Ratio of IgG4-positive plasma cells/IgG-positive cells greater than 40% and the number of IgG4-positive plasma cells greater than 10 per high-powered field.
ο ③ Typical tissue fibrosis, particularly storiform fibrosis, or obliterative phlebitis.
**Diagnosis:**
ο Definite: (1) + (2) + (3)
ο Probable: (1) + (3):
ο Possible: (1) + (2)

Explanatory Note 1: Combination of organ-specific diagnostic criteria *. Patients with a possible or probable diagnosis by comprehensive diagnostic criteria who fulfill the organ-specific criteria for IgG4-RD are regarded as being definite for IgG4-RD. * Diagnostic criteria according to the IgG4-related organ: ① International consensus diagnostic criteria for autoimmune pancreatitis [19], ② IgG4-related lacrimal gland, saliva adenitis diagnostic criteria [20], ③ Diagnostic criteria for IgG4-related kidney disease [24]. Explanatory Note 2: Exclusion diagnosis. (1) It is important to acquire tissue samples from each involved organ to distinguish malignant tumors (e.g., cancer, malignant lymphoma) and similar benign conditions (e.g., Sjögren syndrome, primary sclerosing cholangitis, multicentric Castleman’s disease, secondary retroperitoneal fibrosis, granulomatosis with polyangiitis, sarcoidosis, eosinophilic granulomatosis with polyangiitis). (2) It is important to exclude an infectious- or inflammation-related disease in patients with high fever, highly elevated CRP and neutrophilia. Explanatory Note 3: Pathologic diagnosis. (1) The numbers of IgG4-positive cells are usually more abundant in resected organs and partially enucleated tissue than in tissue samples obtained by needle biopsy or endoscopic biopsy. Thus, it is important to not be too particular about cell number and to provide a precise judgment. (2) Storiform fibrosis is defined as spindle-shaped cells, inflammatory cells and fine collagen fibers forming a flowing arrangement. Obliterative phlebitis is defined as fibrous venous obliteration with inflammatory cells. Both are helpful for a diagnosis of IgG4-RD. ① and ③ without ② can only be applied in a case with poor IgG4 and/or IgG staining. Explanatory Note 4: Steroid reactivity. A steroid trial is not recommended. However, if patients do not respond to initial steroid therapy, the diagnosis should be reconsidered [22,23].
